# Review of tracer kinetic models in evaluation of gliomas using dynamic contrast-enhanced imaging

**DOI:** 10.3389/fonc.2024.1380793

**Published:** 2024-06-14

**Authors:** Jianan Zhou, Zujun Hou, Chuanshuai Tian, Zhengyang Zhu, Meiping Ye, Sixuan Chen, Huiquan Yang, Xin Zhang, Bing Zhang

**Affiliations:** ^1^ Department of Radiology, Nanjing Drum Tower Hospital Clinical College of Nanjing Medical University, Nanjing, China; ^2^ Institute of Medical Imaging and Artificial Intelligence, Nanjing University, Nanjing, China; ^3^ Medical Imaging Center, Department of Radiology, Nanjing Drum Tower Hospital, Affiliated Hospital of Medical School, Nanjing University, Nanjing, China; ^4^ The Jiangsu Key Laboratory of Medical Optics, Suzhou Institute of Biomedical Engineering and Technology, Chinese Academy of Sciences, Suzhou, China

**Keywords:** glioma, dynamic contrast-enhanced, tracer kinetic model, diagnosis, treatment response

## Abstract

Glioma is the most common type of primary malignant tumor of the central nervous system (CNS), and is characterized by high malignancy, high recurrence rate and poor survival. Conventional imaging techniques only provide information regarding the anatomical location, morphological characteristics, and enhancement patterns. In contrast, advanced imaging techniques such as dynamic contrast-enhanced (DCE) MRI or DCE CT can reflect tissue microcirculation, including tumor vascular hyperplasia and vessel permeability. Although several studies have used DCE imaging to evaluate gliomas, the results of data analysis using conventional tracer kinetic models (TKMs) such as Tofts or extended-Tofts model (ETM) have been ambiguous. More advanced models such as Brix’s conventional two-compartment model (Brix), tissue homogeneity model (TH) and distributed parameter (DP) model have been developed, but their application in clinical trials has been limited. This review attempts to appraise issues on glioma studies using conventional TKMs, such as Tofts or ETM model, highlight advancement of DCE imaging techniques and provides insights on the clinical value of glioma management using more advanced TKMs.

## 1 Introduction

**Figure 1:**
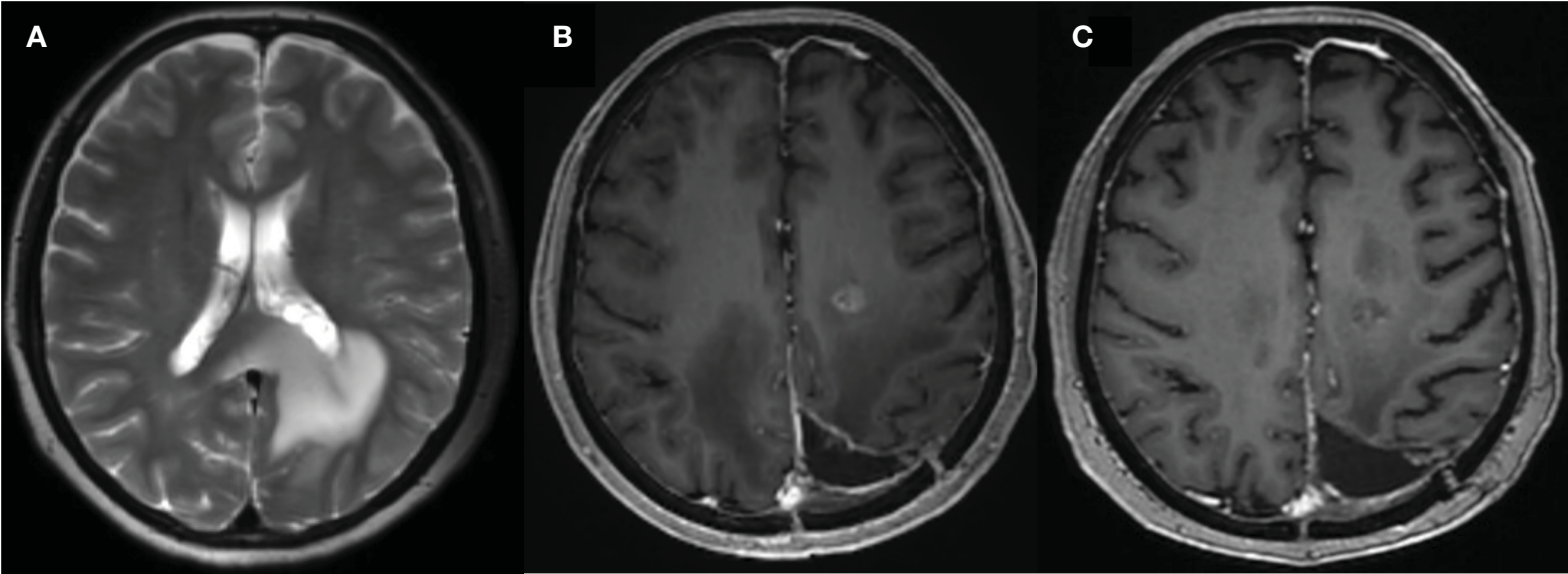
A 59-year-old female with glioma of the left parieto-occipital lobe, treated with surgery and radiation therapy. **(A)** MR image before surgery; **(B)** 10 months after surgery and completion of radiation therapy showed enhancing lesion; **(C)** follow-up MR showed resolution of the lesion.

**Figure 2:**
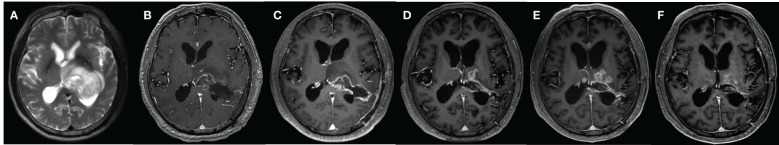
A 67-year-old male with WHO grade 2 glioma of the left basal ganglia and posterior horn of lateral ventricle, treated with surgery, radiotherapy plus concomitant and adjuvant temozolomide. **(A)** MR image before surgery; **(B)** MR image after surgery; **(C)** 1 month after surgery; **(D)** 10 months after surgery; **(E)** 13 months after surgery; **(F)** 15 months after surgery. Follow-up examinations demonstrated the presence of enhancing lesion in 10 months after surgery, which expanded in 13 months and reduced in 15 months.

Glioma originates from the neurostromal cells and is the most common primary tumor of the central nervous system (CNS) (1). It is characterized by wide-spread invasion and angiogenesis, with short median survival duration and high recurrence rate (2). First-line therapy for gliomas consists of radiotherapy, surgery, concomitant chemoradiotherapy and adjuvant chemotherapy with temozolomide (3), while immunotherapies are currently in the pre-clinical and clinical stages of testing (4). The treatment response of glioma is primarily evaluated on the basis of contrast enhanced T1-weighted magnetic resonance imaging (MRI). However, the correlation between changes in enhancement and the treatment response is often confounded by the presence of radiation necrosis, pseudoprogression or pseudoresponse (5–7) (**Figures 1** and **2** for example), thereby warranting more advanced imaging techniques for accurate assessment.

Dynamic contrast enhancement (DCE) imaging is a non-invasive approach that can provide *in vivo* physiological and metabolic information of tissues, and assess microvascular features such as the degree of vascularity and disruption of vascular wall permeability (8–11). DCE imaging data can be analyzed in terms of both semi-quantitative and quantitative parameters. The former includes time-intensity curve (TIC) parameters, such as initial area under the curve (IAUC) and time to peak, which are easy to derive (12) but challenging to reproduce across studies due to differences in data acquisition and subject conditions. DCE imaging data can be quantitatively analyzed using a tracer kinetic model (TKM), a mathematical description of tracer molecular transport within the tissue microenvironment that derives quantitative values of various model parameters pertaining to the tissue status. Several clinical studies have tested DCE imaging for various applications, including glioma assessment (13–17). Most of these studies used Tofts or extended-Tofts model (ETM), which represents early development in DCE imaging. More advanced TKMs have been developed (18, 19), but have received less attention in clinical studies.

In this review, the evaluation of glioma using conventional TKMs, advances in DCE imaging techniques, and the clinical potential of advanced TKMs in glioma management have been discussed.

## 2 Materials and methods

### 2.1 Literature search and selection strategy

**Figure 3:**
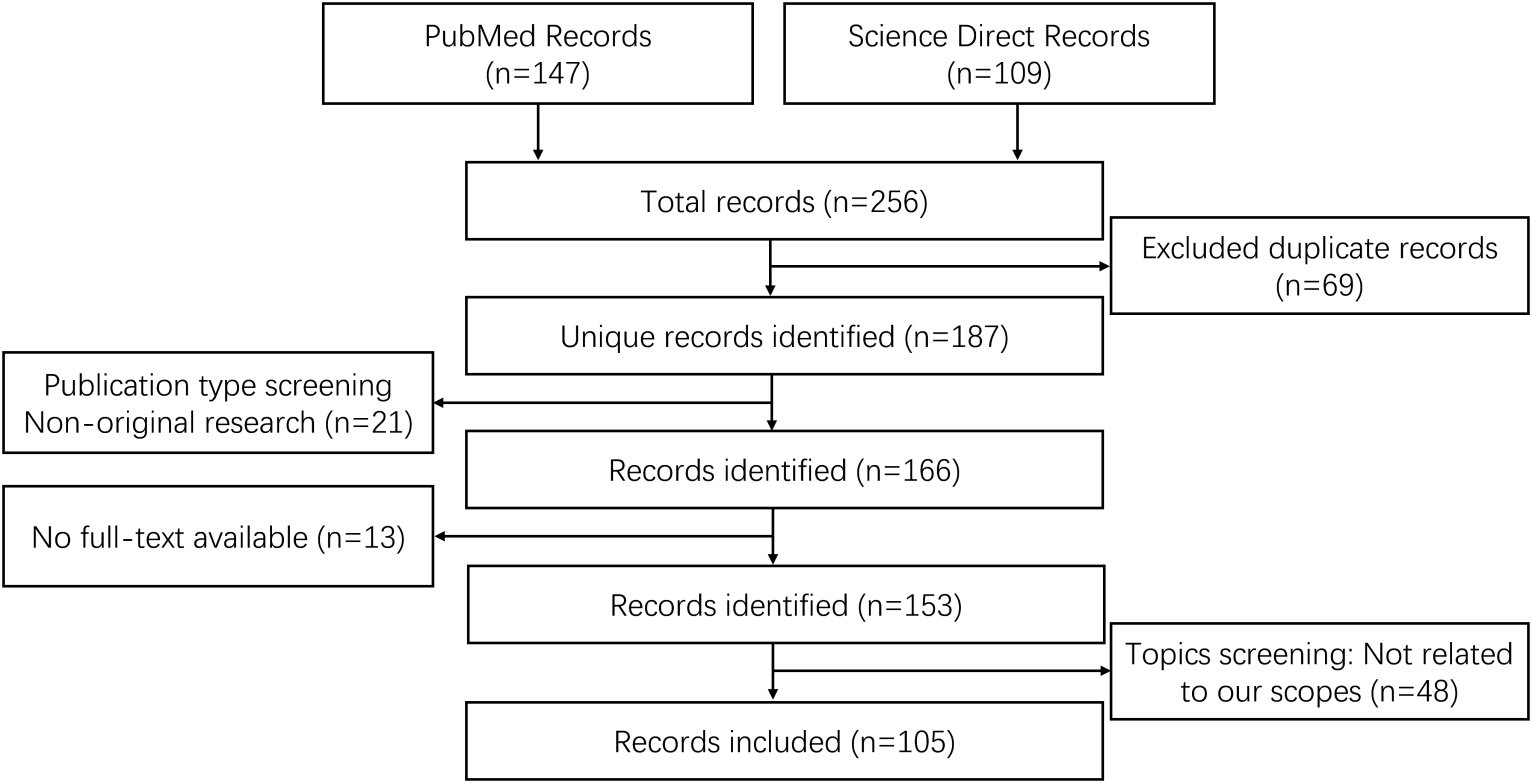
Flowchart of the literature screening process.

We searched for candidate articles describing the different TKMs for gliomas in PubMed and Science Direct databases published between January 1950 and May 2024. The search strategy of key terms used was “((tracer kinetic model OR pharmacokinetic model) OR (Tofts OR generalized kinetic model) OR extended Tofts model OR (two-compartment model OR two-compartment exchange model) OR tissue homogeneity model OR distributed parameter model)) AND (glioma OR glioblastoma) AND (dynamic contrast-enhanced OR DCE)”. The studies were included based on the following inclusion criteria: (1) clinical studies that employed DCE data in patients with gliomas, or experimental animal studies that refer to pathophysiological mechanism of microenvironment; (2) original research published in English with the full text available; and (3) the theoretical basis of tracer kinetic model and its application in glioma diagnosis and evaluation after treatment. The following types of studies were excluded: (1) unrelated or irrelevant studies, such as those that did not employ DCE techniques to investigated gliomas; and (2) studies focusing on other topics that are irrelevant to our research purpose. After a detailed evaluation and screening,105 studies that met our criteria were included and reviewed. The article selection process is shown as a flowchart in **Figure 3**.

### 2.2 Fundamental concepts and primary tracer kinetic models

Tracer kinetic models describe the transport of tracer molecular within the tissue microenvironment. The physical space of the movement of tracer molecules in the tissue is termed as compartment. Typically, there are two well-defined compartments in the field, namely, the compartment of intravascular plasma space (IVPS), and the compartment of extravascular extracellular space (EES). Furthermore, based on the distribution of the tracer, a compartment in TKMs can be categorized as homogeneous or non-homogeneous. For a homogeneous compartment, diffusive resistances are assumed to be zero and the tracer is assumed to move fast and distribute instantaneously and evenly upon arrival in the compartment, which is also termed as a well-mixed compartment. Since the distribution of the tracer is uniform in a well-mixed or homogeneous compartment, the tracer concentration is constant in space and only changes with time (18). TKMs with assumptions on homogeneous or well-mixed compartment are often named as lumped parameter models. In contrast, the tracer concentration varies in a non-homogeneous compartment, and is therefore a function of both space and time. In general, a homogeneous or well-mixed compartment would simplify the modeling process and computation. Thus, the early TKMs were developed based on this assumption. However, the uniform distribution of a tracer in the compartment would depend on rapid movement of tracer molecules or sufficiently long measurement time, neither of which is viable in clinical practice. Therefore, subsequent TKMs introduced a concentration gradient in space to account for the variation in tracer distribution, leading to the assumption of non-homogeneous compartment. Nevertheless, tracer concentration in these models is homogeneous in the radial direction and variable in the axial direction. The meaning of these terms has been summarized in **Table 1**.

**Table 1:** The concept and clinical significance of several terms.

Concept	Meaning
compartment	physical distribution space of tracer in the tissue
well-mixed compartment	tracer distributes evenly throughout compartment
not well-mixed compartment	tracer concentration changes with time and space in compartment
IVPS	intravascular compartment (intravascular plasma space)
EES	interstitial compartment (extravascular extracellular space)
AIF	arterial input function
VIF	vascular input function
relative or normalized parameter	parameter is normalized with respect to contralateral healthy tissue

Primary tracer kinetic models, including Tofts, ETM, Brix’s conventional two-compartment model (Brix), tissue homogeneity model (TH), and distributed parameter model (DP), have been listed in **Table 2**. It is worth pointing out that the notation of a TKM could be different in different studies, and what is introduced here follows largely the notations in earlier review papers on technical aspects of tracer kinetic modeling (18, 19). Tofts model was also named as generalized kinetic (GK) model in (18). The model developed by Brix and coauthors (24, 25) has been denoted as two-compartment exchange model (2CXM) in some literature (19, 35, 36). However, this notation reflects also the fundamental features of other models such as TH and DP, likely leading to confusion in understanding the connection and the difference among these TKMs. To emphasize that the exchange between two-compartments is the common feature of these models, the notation of the model proposed by Brix and coauthors is denoted as conventional two-compartment model (CC or CC2) in (18, 37), or as Brix model in (38). Since ETM is also of two-compartment by nature, confusion could be arisen between CC and ETM. For clarity and simplicity, this presentation adopts the notation of Brix model.

**Table 2:** Summary of primary tracer kinetic models.

Model	References	Compartment	Transport rate parameter	Well-mixed assumption	Independent parameters	Derived parameters
Tofts	[Kety et al. (20),Tofts et al. (21)]	EES	K^trans^	well-mixed	K^trans^, V_e_	K_ep_
ETM	[Tofts et al. (22)]	EES, IVPS	K^trans^	well-mixed	K^trans^, V_e_, V_p_	K_ep_
Brix	[Hayton et al. (23), Brix et al. (24), Brix et al. (25), Larsson et al. (26)]	EES, IVPS	CBF, PS	well-mixed	CBF, PS, V_e_, V_p_	MTT, E
TH	[Johnson (27)], Lawrence et al. (28), Lawrence et al. (29), Lee et al. (30)]	EES, IVPS	CBF, PS	well-mixed EES, not well-mixed IVPS	CBF, PS, V_e_, V_p_	MTT, E
DP	[Bassingthwaighte et al. (31), Larson et al. (32), Koh et al. (33), Koh et al. (34)]	EES, IVPS	CBF, PS	not well-mixed in both compartments	CBF, PS, V_e_, V_p_	MTT, E

For completeness, the equations of the models are given as follows:

Tofts model


$${\text{C}}_{\text{tiss}}\left(t\right)=\text{AIF}⊗{\text{K}}^{\text{trans}}\text{exp}\left(-\frac{{\text{K}}^{\text{trans}}}{v_{e}}t\right)$$

where ⊗ denotes the convolution operator.

Extended-Tofts model (ETM)


$${\text{C}}_{\text{tiss}}\left(t\right)=\text{AIF}⊗\left[{\text{K}}^{\text{trans}}\text{exp}\left(-\frac{{\text{K}}^{\text{trans}}}{v_{e}}t\right)+v_{p}\right]$$

Brix’s conventional two-compartment (Brix) model, Equations 1a–1c


(1a) $${\text{C}}_{\text{tiss}}\left(t\right)=\text{ AIF }⊗{\text{ F}}_{\text{p}}\left[A\;\text{exp}\left(\text{α }t\right)+\left(1-A\right)\text{exp}\left(\text{β }t\right)\right]$$

where


(1b) $$\left(\begin{array}{c} \text{α}\\ \text{β} \end{array}\right)=\frac{1}{2}\left[-\left(\frac{\text{PS}}{{\text{v}}_{\text{p}}}+\frac{\text{PS}}{{\text{v}}_{\text{e}}}+\frac{{\text{F}}_{\text{p}}}{{\text{v}}_{\text{p}}}\right)\pm \sqrt{{\left(\frac{\text{PS}}{{\text{v}}_{\text{p}}}+\frac{\text{PS}}{{\text{v}}_{\text{e}}}+\frac{{\text{F}}_{\text{p}}}{{\text{v}}_{\text{p}}}\right)}^{2}-4\frac{\text{PS}}{{\text{v}}_{\text{e}}}\frac{{\text{F}}_{\text{p}}}{{\text{v}}_{\text{p}}}}\right]$$

(1c) $$\text{A}=\frac{\text{α}+\frac{\text{PS}}{{\text{v}}_{\text{p}}}+\frac{\text{PS}}{{\text{v}}_{\text{e}}}}{\text{α}-\text{β}}\;$$

Distributed parameter (DP) model, Equation 2


(2) $$\begin{array}{c} {\text{C}}_{\text{tiss}}\left(t\right)=\text{AIF }⊗\\ {\text{F}}_{\text{p}}\left\{\begin{array}{l} \text{u}\left(t\right)-\text{u}\left(t-\frac{{\text{v}}_{\text{p}}}{{\text{F}}_{\text{p}}}\right)+\\ \text{u}\left(t-\frac{{\text{v}}_{\text{p}}}{{\text{F}}_{\text{p}}}\right)\{1-\text{exp}\left(-\frac{\text{PS}}{{\text{F}}_{\text{p}}}\right)\left[1+\overset{t-\frac{{\text{v}}_{\text{p}}}{{\text{F}}_{\text{p}}}}{\underset{0}{\int }}\text{exp}\left(-\frac{\text{PS}}{{\text{v}}_{\text{e}}}\text{τ}\right)\sqrt{\frac{\text{PS}}{{\text{v}}_{\text{e}}}\;\frac{\text{PS}}{{\text{F}}_{\text{p}}\;}\frac{1}{\text{τ}}}{\text{ I}}_{1}\left(2\sqrt{\frac{\text{PS}}{{\text{v}}_{\text{e}}}\;\frac{\text{PS}}{{\text{F}}_{\text{p}}}\text{τ }}\right)\text{dτ}\right]\} \end{array}\right\} \end{array}$$

where *u*(*t*) denotes the Heaviside unit-step function and *I*_1_ is the modified Bessel function.

Tissue homogeneity (TH) model, Equation 3


(3) $$\begin{array}{c} {\text{C}}_{\text{tiss}}\left(t\right)=\text{AIF }⊗\\ \;{\text{F}}_{\text{p}}\left\{\begin{array}{c} \left[1-\text{exp}\left(-\frac{\text{PS}}{{\text{F}}_{\text{p}}}\right)\right]\text{exp}\left\{-\frac{{\text{F}}_{\text{p}}}{{\text{v}}_{\text{e}}}\left[1-\text{exp}\left(-\frac{\text{PS}}{{\text{F}}_{\text{p}}}\right)\right]\left(\text{t}-\frac{{\text{F}}_{\text{p}}}{{\text{v}}_{\text{p}}}\right)\right\} \end{array}\right\} \end{array}$$

**Figure 4:**
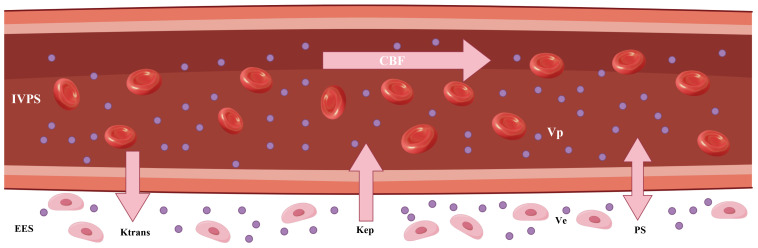
The graphic illustration of various kinetic parameters in tracer kinetic models. IVPS, intravascular compartment (intravascular plasma space); EES, interstitial compartment (extravascular extracellular space); K^trans^, transfer constant; K_ep_, washout rate; CBF, tumor blood flow; V_e_, interstitial volume; V_p_, blood volume; PS, permeability–surface area product.

Tofts assumes that IVPS is significantly smaller than EES, and thus only involves EES. All other models are two-compartment models. The movement of tracer molecules in tissue generally involves intravascular transport and exchange between intravascular and interstitial space. The former reflects blood flow (CBF) and the latter indicates permeability of endothelial wall (PS). Tofts and ETM utilize one parameter (K^trans^) to describe both movements, whereas Brix, TH and DP differentiate between the two and model them separately (24). Tofts, ETM and Brix models assume the compartment to be well-mixed. In the TH model, EES is well-mixed but IVPS is non-homogenous. On the other hand, both compartments are non-homogenous in the DP model. The parameters derived from these models are listed in **Table 2**. The technical details pertaining to these models have been comprehensively discussed in previous reviews (18, 19). The kinetic parameters were graphically presented in **Figure 4**.

### 2.3 Comparison between DCE MRI and DCE CT

As a well-established imaging technique, DCE can be performed by data acquisition on either MRI or CT scanners, followed by image analysis using TKMs (39). Each imaging modality has its merits and demerits. CT is fast in scanning and allows acquisition of images with high resolution in both spatial and temporal space, but at cost of X-ray radiation. MRI is advantageous in better contrast in soft tissue and radiation-free. To reduce radiation in DCE CT, non-uniform acquisition strategy can be adopted to decrease the number of X-ray exposure in the design of DCE CT protocol, with frequent scans during artery phase and less frequent scans during delayed phase. A key difference in terms of DCE language between two modalities lies in the calculation of contrast concentration. Contrast concentration in DCE CT follows simply a linear relationship with CT image signal, whereas the relationship between contrast concentration in DCE MRI with MRI signal is much more complicated, which relates to changes in T1 values of tissue before and after contrast injection. A practical approach to estimating T1 value of tissue is the method of variable flip angles and the computation involves several MR scanning factors such as time of repetition (TR), time of echo (TE), flip angle, homogeneity of B1 field. After deriving contrast concentration from either CT or MR image signals, the analytical process of concentration-time curve will be exactly the same between DCE CT and DCE MRI. For a good appraisal on DCE CT and DCE MRI, interested readers can refer to the review paper (39), where it was shown that data acquisition and analysis were well comparable despite inherent differences in signal production and mechanism of tissue contrast enhancement.

## 3 Application of conventional tracer kinetic models in glioma evaluation

### 3.1 Application in glioma grading

Various studies have analyzed the relationship between DCE imaging parameters and glioma grading (40–49). Santarosa et al. (50) used ETM of DCE MRI in a cohort of 26 glioma patients and demonstrated that V_p_ and K^trans^ differed significantly between low-grade and high-grade gliomas. Using the same model, Zhang et al. (51) found that K^trans^ and V_e_ values calculated in 28 glioma patients based on DCE MRI increased with advanced tumor grade, and significant differences were observed between the low (I and II) and high (III and IV) grade gliomas, as well as between grades II and III. Awasthi et al. (52) applied Tofts model of DCE MRI to 76 glioma patients and showed that K_ep_ and V_e_ could differentiate low-grade from high-grade tumors, although there was no significant correlation between K^trans^ and the expression of MMP-9, which plays a key role in the disruption of the blood-brain barrier (BBB) by degrading extracellular matrix in order to facilitate tumor cell infiltration and metastasis. In contrast, other studies (53, 54) based on DCE MRI have shown that K^trans^ is the most effective parameter for differentiating between glioma grades. These contradictory findings can be attributed to the differences in the extent of BBB disruption among different tumor grades, as well as the different mechanisms underlying BBB disruption in infectious and neoplastic pathologies.

### 3.2 Correlation with immunohistochemical markers

Since the 2021 World Health Organization (WHO) guidelines on the histological classification of central nervous system tumors were published (55), molecular markers have been instrumental in the diagnosis of gliomas. For instance, IDH mutation, 1p/19q co-deletion, TERT promoter mutation, and EGFR gene amplification are key biomarkers used for the classification of diffuse gliomas in adults, and DCE TKMs have been used for evaluating the status of these markers in glioma patients (52, 56–63).

IDH mutation is associated with a survival benefit in glioma patients (64). Wang et al. (65) retrospectively studied the IDH mutation status of 30 patients with low grade gliomas (LGGs) using ETM of DCE MRI, and showed that K^trans^, V_e_ and V_p_ were higher values in the IDH wild-type compared to the IDH mutant LGGs. In contrast, Brendle et al. (66) reported that DCE MRI kinetic parameters derived using the same model did not distinguish between IDH mutant and wild-type astrocytomas. Ahn et al. (67) retrospectively studied the molecular markers in 132 LGG patients using ETM of DCE MRI, and found no significant difference in the DCE kinetic parameters between the IDH mutant and wild-type gliomas.

MGMT promoter methylation predicts favorable prognosis after alkylating drug-based chemotherapy in patients with IDH-wild-type glioblastomas (68). Several studies have evaluated the correlation between MGMT promoter methylation and DCE kinetic parameters in gliomas (69–71). Ahn et al. (67) showed that K^trans^, V_e_ and V_p_ values derived using ETM of DCE MRI were significantly lower in MGMT methylated LGGs than in the unmethylated counterparts. Another study (70) using the Tofts model of DCE MRI showed that K^trans^ values were significantly higher in the MGMT methylated tumors, while K_ep_ and V_e_ showed no significant difference between gliomas with methylated and unmethylated MGMT. Hilario et al. (69) analyzed 49 glioma patients using ETM of DCE MRI, and did not detect any significant differences in the DCE kinetic parameters of the MGMT methylated and non-methylated tumors. Zhang et al. (71)further showed that gliomas with non-methylated MGMT had higher V_e_ and K^trans^ values with ETM of DCE MRI than those with methylated MGMT.

### 3.3 Differential diagnosis of glioma, PCNSL, and metastasis

Due to differences in clinical treatment and prognosis, it is critical to distinguish between primary central nervous system lymphoma (PCNSL), high-grade gliomas (HGGs), and metastatic glioma (72–79). Xi et al. (80) used Tofts model of DCE MRI to retrospectively analyze 8 cases of PCNSL, 21 cases of HGGs and 6 cases of metastasis, and detected significantly higher K^trans^ and V_e_ in the PCNSL tumors compared to HGGs and metastatic tumors. However, Kickingereder et al. (81) did not observe any significant difference in the V_e_ values of PCNSL and GBM using ETM of DCE MRI. Lin et al. (82) showed that PCNSL had higher values of K^trans^ using ETM of DCE MRI compared to GBM, although the difference was not statistically significant. Jin et al. (83) used ETM of DCE MRI to show that K^trans^ had the highest specificity and sensitivity in differentiating between GBM, PCNSL, and metastasis. Kang et al. (84) calculated the V_p_ in glioblastomas and PCNSL through ETM of DCE MRI, and found that the values were significantly higher in the former. In contrast, Abe et al. (43) used ETM of DCE MRI to analyze 29 lesions (including glioma, metastatic tumor and lymphoma) and found that V_p_ was not helpful in differentiating PCNSL from GBM.

### 3.4 Evaluation of treatment response

All patients with glioblastoma eventually relapse. It is challenging to differentiate relapsed tumor from other treatment-related changes during the follow-up of glioma patients (85–92). Thomas et al. (93) found that V_p_ and K^trans^ measurements were lower in glioblastoma patients with pseudoprogression compared to those with relapsed lesions using ETM of DCE MRI. Yun et al. (94) also applied ETM of DCE MRI to differentiate false progression from true progression, and found that K^trans^ and V_e_ were significantly higher in the latter, whereas V_p_ value was similar for both types of lesions. Jing et al. (95) applied the Tofts model of DCE MRI to a retrospective analysis of 51 patients with new enhancement lesions after standard radiotherapy and chemotherapy after surgical resection, and found no significant difference in V_e_ and K_ep_ between the pseudoprogression group and the recurrence group.

## 4 Assessment of gliomas using advanced tracer kinetic models

### 4.1 Glioma grading

Jain et al. (96, 97) investigated glioma grading using perfusion CT (PCT) with the TH model (27) to estimate permeability and blood volume. While the rCBV increased more than the PS in LGGs, grade 3 and especially grade 4 gliomas showed a greater increase in PS compared to rCBV. The rate of change in the rCBV/PS ratio may correlate to changes occurring at the tumor microvasculature level. Both PS and CBV were higher in the HGGs compared to the LGGs. Furthermore, PS can also be used to differentiate WHO grade 3 glioma from grade 4 glioma. Tietze et al. (35) presented a Bayesian framework for parameter optimization of tracer kinetic modeling and compared the Brix model and ETM in the grading of 42 untreated glioma patients. Brix-derived V_p_ showed the best diagnostic performance with AUC of 0.97, followed by ETM-derived K^trans^ with an AUC of 0.92. However, the diagnostic performance of permeability parameter was low, with AUC of 0.57.

### 4.2 Monitoring of treatment response

#### 4.2.1 *In vivo* human studies

Jensen et al. (98) correlated parameters derived from a gamma capillary transit time model of DCE MRI, which is based on the distributed capillary approximated TH model (99, 100), with molecular markers of hypoxia, vascularity, proliferation, and progression-free and overall survival (OS) in a cohort of 18 glioma patients. The derived parameters included tumor blood flow (CBF), extraction fraction (E), permeability–surface area product (PS), transfer constant (K^trans^), washout rate (K_ep_), interstitial volume (V_e_), blood volume (V_p_), capillary transit time (tc), and capillary heterogeneity ( $\alpha^{-1}$ ). The study showed that $\alpha^{-1}$ , tc, K_ep_, and V_p_ were correlated with HIF-1α and VEGF expression, whereas V_e_ and $\alpha^{-1}$ were correlated with OS. The other imaging markers were not helpful in predicting OS. In particular, none of the blood flow and permeability parameters (K^trans^, CBF, E, PS) showed any correlation with patient outcome.

Lundemann et al. (101) explored the feasibility of predicting tumor recurrence using multi-modal imaging based on DCE MRI at the pre-radiotherapy stage in a cohort of 16 glioblastoma patients using Brix-derived DCE parameters. The median MTT, V_p_, V_e_ and PS derived from scans prior to chemoradiotherapy showed differences between recurring and non-recurring voxels. Henriksen et al. (102) investigated the diagnostic value of Brix-derived parameters of DCE MRI for short-term disease progression in 60 anaplastic astrocytoma and glioblastoma patients with suspected recurrence and evaluable outcome within 6 months of follow-up as determined by histopathology, MRI findings or clinical decision. The blood volume and vascular permeability were significantly higher in the progressive lesions compared to the non-progressive lesions. ROC analysis showed that blood flow and blood volume had AUC values of 0.76 and 0.78 respectively, which were higher than that of vascular permeability (0.68).

Larsen et al. (103) utilized Brix of DCE MRI to differentiate tumor recurrence from radiation necrosis in 19 glioma patients following surgery and radiation therapy, and demonstrated that an empirical threshold of 2 ml/100 g for blood volume allowed detection of regressing lesions with sensitivity and specificity of 100% each. In comparison, neither blood flow nor permeability parameter could discriminate between regressing and progressing lesions.

#### 4.2.2 *Ex vivo* animal studies

Kiser et al. (36) applied the Brix model of DCE MRI to evaluate test-retest repeatability and tumor response of a murine glioblastoma model at 7 T to a combination therapy of bevacizumab and fluorouracil. Test-retest experiments demonstrated that there was no significant difference between the scans in terms of the median values of parameters, except for K^trans^. The compartmental volume fractions (V_e_ and V_p_) remained more consistent between scans while the vascular functional parameters (CBF and PS) showed noticeable increase in values, likely due to physiological changes in the tumor between scans. The tumor volume in the control and treated groups did not differ significantly at any time point, which corresponded to similar tracer kinetic parameters in both groups.

Yeung et al. (104) investigated the efficacy of CT perfusion imaging as an early biomarker of the response to stereotactic radiosurgery in a rat glioma model using the TH model (27). Rats with orthotopic C6 glioma tumors received either mock irradiation or stereotactic radiosurgery delivered by Helical Tomotherapy. The responders to stereotactic radiosurgery showed lower relative CBV, and PS on day 7 post-stereotactic radiosurgery when compared to controls and non-responders. Relative CBV and PS on day 7 were correlated with the OS, and predicted survival with 92% accuracy.

### 4.3 Correlation to immunohistochemical markers

Li et al. (105) applied the DP model of DCE MRI to a dataset consisting of 55 glioma patients to assess glioma isocitrate dehydrogenase (IDH) mutation. The IDH-mutant gliomas showed significantly lower CBF, PS, V_p_, E and V_e_ compared to the IDH-wildtype gliomas. V_p_ exhibited the best performance in differentiating between IDH-mutant and IDH-wildtype gliomas (AUC=0.92), followed by PS (AUC=0.82) and E (AUC=0.8). Furthermore, the Tofts parameters K^trans^ and V_e_ were lower for the IDH-mutant gliomas, and no significant difference was observed in K_ep_. The AUCs of K^trans^, V_e_, and K_ep_ were 0.69, 0.79, and 0.55 respectively. These findings suggested that IDH-mutant gliomas have lower vascularity, vessel permeability and blood flow compared to IDH-wildtype gliomas, which may explain the better outcomes in patients with IDH-mutated versus IDH-wildtype gliomas.

## 5 Discussion

### 5.1 Advantage of advanced TKMs over conventional ones

The fifth edition of the WHO Classification of CNS Tumors (WHO CNS5), published in 2021, builds on the fourth edition updated in 2016 and the work of the Consortium to Inform Molecular and Practical Approaches to CNS Tumor Taxonomy, further advancing the role of molecular diagnostics in the classification of CNS tumors, but remains rooted in other established methods for assessing tumor characteristics, including histology and immunohistochemistry (55). Besides, the existing evaluation of glioma features is still based on pathological biopsy as the gold standard. Due to the high heterogeneity of gliomas and the influence of non-uniform sampling error of tumors, the diagnostic accuracy is limited by the subjective judgment of pathologists. In 2021 WHO CNS5, EGFR amplification is commonly seen in IDH wild-type gliomas. EGFR is a tyrosine kinase receptor regulating cell proliferation and differentiation by interacting with epidermal growth factor (EGF) and tumor growth factor-α (TGF-α), and can reflect microvascular proliferation in tumors (106). With the development of MRI technologies, more abundant characteristics of tumor microenvironment have been provided for the non-invasive diagnosis of gliomas. DCE MRI is sensitive to the destruction of the blood-brain barrier (BBB) and is closely related to microvascular proliferation and vascular wall permeability, which can effectively evaluate tumor angiogenesis and obtain various kinetic parameters reflecting tissue microcirculation function (9).

The focus of this review was to reveal the characteristics of the immune microenvironment of gliomas based on the conventional and advanced TKMs of DCE imaging, and to provide a non-invasive method for the diagnosis and treatment response evaluation of gliomas. Most studies on glioma assessment with DCE imaging have used Tofts or ETM for image postprocessing and data analysis, and the results show considerable variability. For instance, some studies (50, 107) have reported that ETM-derived V_p_ is a useful imaging parameter that can discriminate between LGG and HGG, whereas one study (51) showed that V_e_ and not V_p_ differed significantly between LGG and HGG. Likewise, Arevalo-Perez et al. (42) showed that V_p_ had the best discriminatory power in glioma grading, whereas Jung et al. (44) reported that K^trans^ was the most significant pharmacokinetic parameter. ETM-derived parameters can also distinguish IDH-mutant gliomas from IDH wildtype gliomas (65), although some studies (66, 67) have reported contradictory findings. While ETM_V_p_ was reported to be significantly higher for glioblastomas compared to PCNSL in one study (84), another study (43) showed that it could not differentiate between the two lesions. Furthermore, K^trans^ derived from the Tofts model was demonstrated to be a promising discriminatory biomarker for LGG relative to HGG (41, 108), although Awasthi et al. (52) found that K^trans^ was not significantly different between LGG and HGG. The variations in the results across the different studies using Tofts or ETM, and the inconsistent performance of K^trans^ in particular, have been reported in earlier reviews (13, 14).

Quantitative imaging biomarkers alliance (QIBA) has recommended ETM for analyzing brain tumors through DCE imaging (109). Both imaging hardware and DCE tracer kinetic modeling have undergone significant advances over the years, thereby allowing acquisition of DCE images with higher temporal resolution, better signal-to-noise ratio, wider brain coverage and increased spatial resolution, and enabling separate quantification of CBF and PS. The advantages of a DP model over the Tofts model have been demonstrated in the assessment of the IDH mutation status in glioma (105). Recent test-retest experiments using Brix (36) showed that only the median K^trans^ was significantly different between scans, which might justify the separate modeling of intravascular transport and exchange between intra- and extra-vascular space in Brix, instead of mixed modeling using one transport rate parameter (K^trans^). HIF-1α expression is significantly associated with HGGs (110) and is an excellent biomarker for glioma grading (111). Furthermore, gliomas are typically characterized by a marked increase in the formation of blood vessels with abnormal blood flow and increased vessel permeability. In fact, blood volume is a promising imaging biomarker for glioma grading (42, 50, 107). As expected, V_p_ derived using a DP model showed good correlation with HIF-1α expression (r= 0.747, P=0.043) (108). However, low correlation was observed between HIF-1α expression and ETM-derived V_p_ (r= 0.149, P=0.219) in another study (111).

**Figure 5:**
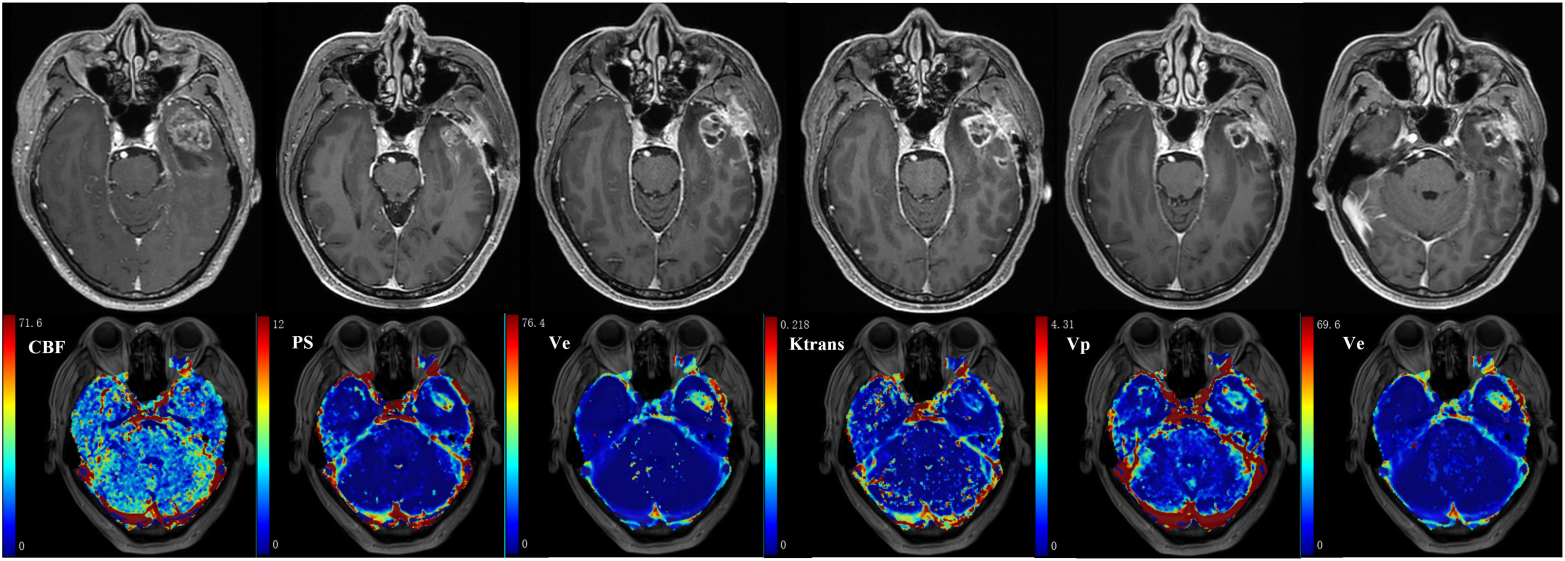
59-year-old male with glioblastoma of the left temporal lobe. Row 1: follow-up MR examinations after surgery showed the evolution of enhancing lesion. Row 2: parametric maps of blood flow CBF, vessel permeability PS and fractional volume of interstitial space V_e_ as derived using DP. Parametric maps of volume transfer constant K^trans^, V_p_ and V_e_ as derived using ETM.

Physiological interpretation of K^trans^ has been ambiguous in clinical trials using Tofts or ETM, and is generally described as a volume transfer constant that reflects vessel wall permeability (13, 14). Several theoretical descriptions have been provided to interpret K^trans^ as a combination of blood flow and vessel wall permeability (112, 113). Separate measurement of CBF and PS has been well addressed in nuclear medicine (114), which is crucial to the understanding of tissue hemodynamics, and has spurned the development of more advanced DCE TKMs. In **Figure 5**, the parametric maps of ETM and DP for a glioblastoma (GBM) patient after surgery have been compared. The follow-up MR images in Row 1 demonstrated the evolution of enhancing lesion. DP-derived parameters in Row 2 showed that the enhancing lesion was characterized by reduced blood flow, increase in PS and marginal increase in V_e_ compared to the contralateral tissue. ETM-derived parameters in Row 2 manifested as reduced K^trans^, marginal increase in V_p_, and increase in V_e_. As previously reported (24), V_e_ in the brain tumor reflects the transfer of tracer molecules between intravascular and interstitial space due to insufficient leakage from the intravascular space and attainment of steady levels in the interstitial space during the insufficient scanning period. Hence, ETM and DP illustrated similar pattern in vessel wall permeability in this case. Nonetheless, the lower value of K^trans^, which is generally explained as reduced vessel wall permeability, may be indicative of reduced blood flow for this case.

**Figure 6:**
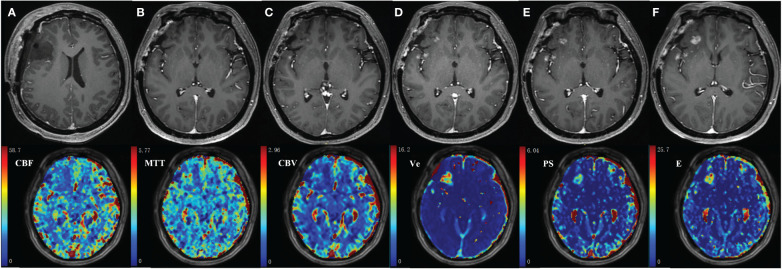
35-year-old female with WHO grade 4 glioma of the right frontal lobe. Postoperative pathology confirmed the enhanced lesion as radiation necrosis (RN). Row 1: follow-up MR examinations after surgery showed the presence and evolution of enhancing lesion. **(A)** MR image after the first surgery; **(B)** 30 months after surgery; **(C)**31 months after surgery; **(D)**33 months after surgery; **(E)**35 months after surgery; **(F)**37 months after surgery. Row 2: parametric maps of blood flow CBF, mean transit time MTT, fractional volume of intravascular space CBV, fractional volume of interstitial space V_e_, vessel permeability PS, and extraction ratio E as derived using DP model.

The MR images of WHO grade 4 glioma at the right frontal lobe treated with surgery and chemoradiotherapy are shown in **Figure 6**. Small enhancing lesions appeared in the 31^th^ month of follow-up after surgery and continued to grow. DCE scan was performed in the 37^th^ month of follow-up, and analyzed using the distributed parameter model. The parametric maps are shown in the second row, which indicate reduced blood flow and increased vessel permeability in the lesion compared to the contralateral tissue. The patient underwent a second surgery, and histopathological analysis revealed necrotic foci with no evidence of recurrent tumor.

In the traditional WHO classification of tumors, each tumor type has a unique WHO grading, such as anaplastic astrocytoma, which is automatically assigned to WHO grade III. The latest 2021 WHO Classification of CNS Tumors emphasizes the combination of histological grades and molecular markers, such as Glioblastoma, IDH-wild type; Astrocytoma, IDH-mutant; or Oligodendroglioma, IDH-mutant & 1p/19q codeleted. IDH mutant astrocytoma expands from the CNS in WHO grade 2–4. However, most of the current studies are based on the 2016 WHO Classification, lacking specific tumor pathological classification diagnosis (**Table 3**). Therefore, future studies need to use more advanced MRI technologies for further comprehensive diagnosis and stratified reporting of gliomas.

**Table 3:** Application of tracer kinetic models in glioma grading and molecular markers according to WHO classification.

Model	References	Tasks	WHO Classification
Tofts	Zhang et al. (2012) (51), Awasthi et al. (2012) (52)	grading	2007
	Ahn et al. (2014) (70)	MGMT methylation	2007
	Jia et al. (2021) (41), Zhao et al. (2017) (53)	grading	2016
ETM	Santarosa et al. (2016) (50)	grading	2016
	Wang et al. (2020) (65), Brendle et al. (2018) (66)	IDH mutation	2016
	Ahn et al. (2023) (67)	IDH mutation, EGFR, MGMT and TERT	2016
	Hilario et al. (2019) (69)	IDH mutation, ATRX, and MGMT	2016
	Zhang et al. (2020) (71)	IDH mutation, MGMT and TERT	2016
	Anzalone et al. (2018) (40)	grading, IDH mutation and 1p19q codeletion	2016
TH	Jain et al. (2008,2015) (96, 97)	grading	2007
DP	Li et al. (2020) (105)	IDH mutation	2016

### 5.2 Standardization of DCE data acquisition and postprocessing

Despite limited research on advanced DCE imaging TKMs for glioma, the differences across studies are obvious. Brix-derived blood volume has shown high discriminatory ability for regressing lesions, whereas blood flow and permeability parameters showed fairly low discriminating power in the differential diagnosis of tumor recurrence (103). In contrast, a recent study (102) demonstrated similar ability of blood flow and blood volume in differentiating progressive lesions from non-progressive lesions, and significantly higher permeability in the former. Multiple factors can contribute to these discrepancies, such as DCE imaging protocols, data post-processing, scanners, operators in data processing, sample size and patient characteristics, etc. QIBA has made the following recommendations to standardize DCE MR imaging protocol (115): (1) 3D T1-weighted GRE sequence, (2) variable flip angle (VFA) method to estimate T1 map and contrast agent concentration, (3) same sequence for pre-contrast VFA scan and post-contrast dynamic scan, (4) temporal resolution not lower than 4 s in most cases, (5) sufficiently long scanning duration of about 6 min for permeability measurement. The DCE protocols used in earlier studies often deviated from QIBA recommendations, whereas the more recent studies (102) have protocols closer to QIBA guidelines. DCE imaging data is largely analyzed using commercial software programs that differ in algorithm implementation, which may result in significant differences in the estimated DCE parameters (116). When analyzing DCE images, it is critical to accurately determine the contrast concentration from image intensities. The relationship between image intensity and concentration can be non-linear, particular for MRI. In some software programs, linear assumption between change in signal intensity and gadolinium concentration is used to directly derive the contrast concentration-time curve from signal intensity-time curve (42). The choice of vascular input function (VIF) also affects the estimated values of DCE parameters. It would be ideal to select VIF from DCE images for each patient. Some software programs use population VIF or empirical VIF, as summarized in the QIBA profile (109). Standardization of imaging protocol and postprocessing procedure is crucial in making DCE reproducible and bringing forward the technology into clinical practice.

### 5.3 Cross-validation in DCE studies

ROC curve analysis is a useful tool for characterizing the differential diagnostic ability of potential biomarkers, which can be quantified by calculating the area under the ROC curve (AUC), or by finding an optimal threshold from the ROC curve and determining the associated sensitivity, specificity and accuracy. Most studies on DCE imaging-based glioma assessment directly applied the ROC curve method to the total data, and reported the performance of DCE parameters. In clinical practice, however, the imaging data is invariably subjected to different sources of perturbations. Therefore, the resulting diagnostic metrics could be biased towards best matching pattern in the current data, and their performance could be significantly different when applied to new data. This phenomenon is called overfitting in machine learning (117) and is particularly acute when the sample size is small, which is often the case in glioma studies. This issue is commonly resolved by cross-validation, which is typically performed by dividing data into the training set and validation set, and applying the information derived from the former to the latter. K-fold cross-validation is the most frequently used cross-validation method in machine learning. However, the leave-one-out cross-validation method is recommended for glioma studies since the cohort size is usually small. Shao et al. (38) used this method to assess the parameters of DCE imaging in a small cohort of cervical cancer patients. The following steps were used: (1) one data was left out, and the remaining data was used as the training set to build a model and make prediction on the excluded data, (2) the previous step was repeated for each data, and (3) the differential diagnostic ability was quantified by summarizing all predicted values. In the current DCE glioma studies, performance quantification without cross-validation has resulted in variation between the results of different research groups.

## 6 Conclusion

DCE imaging is effective in glioma grading and therapeutic effect monitoring, and its parameters are potential imaging markers for glioma diagnosis. However, the discrepancies between the findings in different studies warrant further improvement and validation of this technique with standardization of protocol design, and data post-processing in multi-center and large cohorts. Advanced DCE imaging techniques that allow separate modeling of blood flow and vessel permeability have advantages over conventional counterparts, although more studies are needed to ascertain the clinical value.

## Author contributions

JZ: Conceptualization, Formal analysis, Funding acquisition, Investigation, Writing – original draft, Writing – review & editing. ZH: Conceptualization, Formal analysis, Methodology, Software, Writing – original draft, Writing – review & editing. CT: Conceptualization, Formal analysis, Writing – original draft, Writing – review & editing. ZZ: Conceptualization, Formal analysis, Writing – original draft, Writing – review & editing. MY: Conceptualization, Formal analysis, Writing – original draft, Writing – review & editing. SC: Conceptualization, Writing – original draft, Writing – review & editing. HY: Conceptualization, Writing – original draft, Writing – review & editing. XZ: Conceptualization, Funding acquisition, Methodology, Supervision, Writing – original draft, Writing – review & editing. BZ: Funding acquisition, Supervision, Writing – original draft, Writing – review & editing.

## References

[1] Ostrom QT Gittleman H Truitt G Boscia A Kruchko C Barnholtz-Sloan JS. CBTRUS statistical report: primary brain and other central nervous system tumors diagnosed in the United States in 2011–2015. Neuro Oncol. (2018) 20:iv1–iv86. doi: 10.1093/neuonc/noy131 30445539 PMC6129949

[2] Lapointe S Perry A Butowski NA. Primary brain tumours in adults. Lancet. (2018) 392:432–46. doi: 10.1016/S0140-6736(18)30990-5 30060998

[3] Weller M van den Bent M Preusser M Le Rhun E Tonn JC Minniti G. EANO guidelines on the diagnosis and treatment of diffuse gliomas of adulthood. Nat Rev Clin Oncol. (2021) 18:170–86. doi: 10.1038/s41571-020-00447-z PMC7904519 33293629

[4] Lim M Xia Y Bettegowda C Weller M. Current state of immunotherapy for glioblastoma. Nat Rev Clin Oncol. (2018) 15:422–42. doi: 10.1038/s41571-018-0003-5 29643471

[5] Dhermain FG Hau P Lanfermann H Jacobs AH van den Bent MJ. Advanced MRI and PET imaging for assessment of treatment response in patients with gliomas. Lancet Neurol. (2010) 9:906–20. doi: 10.1016/S1474-4422(10)70181-2 20705518

[6] Zikou A Sioka C Alexiou GA Fotopoulos A Voulgaris S Argyropoulou MI. Radiation necrosis, pseudoprogression, pseudoresponse, and tumor recurrence: imaging challenges for the evaluation of treated gliomas. Contrast Media Mol Imaging. (2018) 2018:6828396. doi: 10.1155/2018/6828396 30627060 PMC6305027

[7] Fink J Born D Chamberlain MC. Pseudoprogression: relevance with respect to treatment of high-grade gliomas. Curr Treat Options Oncol. (2011) 12:240–52. doi: 10.1007/s11864-011-0157-1 21594589

[8] Heye AK Thrippleton MJ Armitage PA Valdés Hernández MDC Makin SD Glatz A. Tracer kinetic modelling for DCE-MRI quantification of subtle blood-brain barrier permeability. Neuroimage. (2016) 125:446–55. doi: 10.1016/j.neuroimage.2015.10.018 PMC4692516 26477653

[9] Heye AK Culling RD Valdés Hernández MDC Thrippleton MJ Wardlaw JM. Assessment of blood-brain barrier disruption using dynamic contrast-enhanced MRI. A systematic review. NeuroImage Clin. (2014) 6:262–74. doi: 10.1016/j.nicl.2014.09.002 PMC4215461 25379439

[10] Nielsen T Wittenborn T Horsman MR. Dynamic contrast-enhanced magnetic resonance imaging (DCE-MRI) in preclinical studies of antivascular treatments. Pharmaceutics. (2012) 4:563–89. doi: 10.3390/pharmaceutics4040563 PMC3834929 24300371

[11] Eilaghi A Yeung T d’Esterre C Bauman G Yartsev S Easaw J. Quantitative perfusion and permeability biomarkers in brain cancer from tomographic CT and MR images. biomark Cancer. (2016) 8:47–59. doi: 10.4137/BIC.S31801 27398030 PMC4933536

[12] Narang J Jain R Arbab AS Mikkelsen T Scarpace L Rosenblum ML. Differentiating treatment-induced necrosis from recurrent/progressive brain tumor using nonmodel-based semiquantitative indices derived from dynamic contrast-enhanced T1-weighted MR perfusion. Neuro Oncol. (2011) 13:1037–46. doi: 10.1093/neuonc/nor075 PMC3158013 21803763

[13] Zahra MA Hollingsworth KG Sala E Lomas DJ Tan LT. Dynamic contrast-enhanced MRI as a predictor of tumour response to radiotherapy. Lancet Oncol. (2007) 8:63–74. doi: 10.1016/S1470-2045(06)71012-9 17196512

[14] O’Connor JPB Jackson A Parker GJM Roberts C Jayson GC. Dynamic contrast-enhanced MRI in clinical trials of antivascular therapies. Nat Rev Clin Oncol. (2012) 9:167–77. doi: 10.1038/nrclinonc.2012.2 22330689

[15] Fouke SJ Benzinger T Gibson D Ryken TC Kalkanis SN Olson JJ. The role of imaging in the management of adults with diffuse low grade glioma: A systematic review and evidence-based clinical practice guideline. J Neurooncol. (2015) 125:457–79. doi: 10.1007/s11060-015-1908-9 26530262

[16] Guida L Stumpo V Bellomo J van Niftrik CHB Sebök M Berhouma M. Hemodynamic imaging in cerebral diffuse glioma-part A: concept, differential diagnosis and tumor grading. Cancers (Basel). (2022) 14:1432. doi: 10.3390/cancers14061432 35326580 PMC8946242

[17] Hirschler L Sollmann N Schmitz-Abecassis B Pinto J Arzanforoosh F Barkhof F. Advanced MR techniques for preoperative glioma characterization: part 1. J Magn Reson Imaging. (2023) 57:1655–75. doi: 10.1002/jmri.28662 PMC10946498 36866773

[18] Koh TS Bisdas S Koh DM Thng CH. Fundamentals of tracer kinetics for dynamic contrast-enhanced MRI. J Magn Reson Imaging. (2011) 34:1262–76. doi: 10.1002/jmri.22795 21972053

[19] Sourbron SP Buckley DL. Tracer kinetic modelling in MRI: estimating perfusion and capillary permeability. Phys Med Biol. (2012) 57:R1–33. doi: 10.1088/0031-9155/57/2/R1 22173205

[20] Kety SS. The theory and applications of the exchange of inert gas at the lungs and tissues. Pharmacol Rev. (1951) 3:1–41. 14833874

[21] Tofts PS Kermode AG. Measurement of the blood-brain barrier permeability and leakage space using dynamic MR imaging. 1. Fundamental concepts. Magn Reson Med. (1991) 17:357–67. doi: 10.1002/mrm.1910170208 2062210

[22] Tofts PS. Modeling tracer kinetics in dynamic Gd-DTPA MR imaging. J Magn Reson Imaging. (1997) 7:91–101. doi: 10.1002/jmri.1880070113 9039598

[23] Hayton P Brady M Tarassenko L Moore N. Analysis of dynamic MR breast images using a model of contrast enhancement. Med Image Anal. (1997) 1:207–24. doi: 10.1016/s1361-8415(97)85011-6 9873907

[24] Brix G Bahner ML Hoffmann U Horvath A Schreiber W. Regional blood flow, capillary permeability, and compartmental volumes: measurement with dynamic CT–initial experience. Radiology. (1999) 210:269–76. doi: 10.1148/radiology.210.1.r99ja46269 9885619

[25] Brix G Kiessling F Lucht R Darai S Wasser K Delorme S. Microcirculation and microvasculature in breast tumors: pharmacokinetic analysis of dynamic MR image series. Magn Reson Med. (2004) 52:420–9. doi: 10.1002/mrm.20161 15282828

[26] Larsson HBW Courivaud F Rostrup E Hansen AE. Measurement of brain perfusion, blood volume, and blood-brain barrier permeability, using dynamic contrast-enhanced T(1)-weighted MRI at 3 tesla. Magn Reson Med. (2009) 62:1270–81. doi: 10.1002/mrm.22136 19780145

[27] Johnson JA Wilson TA. A model for capillary exchange. Am J Physiol. (1966) 210:1299–303. doi: 10.1152/ajplegacy.1966.210.6.1299 5923068

[28] St Lawrence KS Lee TY. An adiabatic approximation to the tissue homogeneity model for water exchange in the brain: I. Theoretical derivation. J Cereb Blood Flow Metab. (1998) 18:1365–77. doi: 10.1097/00004647-199812000-00011 9850149

[29] St Lawrence KS Lee TY. An adiabatic approximation to the tissue homogeneity model for water exchange in the brain: II. Experimental validation. J Cereb Blood Flow Metab. (1998) 18:1378–85. doi: 10.1097/00004647-199812000-00012 9850150

[30] Lee T-Y Purdie TG Stewart E. CT imaging of angiogenesis. Q J Nucl Med. (2003) 47:171–87. 12897709

[31] Bassingthwaighte JB. A concurrent flow model for extraction during transcapillary passage. Circ Res. (1974) 35:483–503. doi: 10.1161/01.res.35.3.483 4608628 PMC3077802

[32] Larson KB Markham J Raichle ME. Tracer-kinetic models for measuring cerebral blood flow using externally detected radiotracers. J Cereb Blood Flow Metab. (1987) 7:443–63. doi: 10.1038/jcbfm.1987.88 3611204

[33] Koh TS Cheong LH Hou Z Soh YC. A physiologic model of capillary-tissue exchange for dynamic contrast-enhanced imaging of tumor microcirculation. IEEE Trans BioMed Eng. (2003) 50:159–67. doi: 10.1109/TBME.2002.807657 12665029

[34] Koh TS Thng CH Lee PS Hartono S Rumpel H Goh BC. Hepatic metastases: *in vivo* assessment of perfusion parameters at dynamic contrast-enhanced MR imaging with dual-input two-compartment tracer kinetics model. Radiology. (2008) 249:307–20. doi: 10.1148/radiol.2483071958 18695207

[35] Tietze A Nielsen A Klærke Mikkelsen I Bo Hansen M Obel A Østergaard L. Bayesian modeling of Dynamic Contrast Enhanced MRI data in cerebral glioma patients improves the diagnostic quality of hemodynamic parameter maps. PloS One. (2018) 13:e0202906. doi: 10.1371/journal.pone.0202906 30256797 PMC6157834

[36] Kiser K Zhang J Das AB Tranos JA Wadghiri YZ Kim SG. Evaluation of cellular water exchange in a mouse glioma model using dynamic contrast-enhanced MRI with two flip angles. Sci Rep. (2023) 13:3007. doi: 10.1038/s41598-023-29991-1 36810898 PMC9945648

[37] Lu Y Peng W Song J Chen T Wang X Hou Z. On the potential use of dynamic contrast-enhanced (DCE) MRI parameters as radiomic features of cervical cancer. Med Phys. (2019) 46:5098–109. doi: 10.1002/mp.13821 31523829

[38] Shao J Zhang Z Liu H Song Y Yan Z Wang X. DCE-MRI pharmacokinetic parameter maps for cervical carcinoma prediction. Comput Biol Med. (2020) 118:103634. doi: 10.1016/j.compbiomed.2020.103634 32174312

[39] O’Connor JPB Tofts PS Miles KA Parkes LM Thompson G Jackson A. Dynamic contrast-enhanced imaging techniques: CT and MRI. Br J Radiol. (2011) 84 Spec No 2:S112–120. doi: 10.1259/bjr/55166688 PMC3473907 22433822

[40] Anzalone N Castellano A Cadioli M Conte GM Cuccarini V Bizzi A. Brain gliomas: multicenter standardized assessment of dynamic contrast-enhanced and dynamic susceptibility contrast MR images. Radiology. (2018) 287:933–43. doi: 10.1148/radiol.2017170362 29361245

[41] Jia L Wu X Wan Q Wan L Jia W Zhang N. Effects of artery input function on dynamic contrast-enhanced MRI for determining grades of gliomas. Br J Radiol. (2021) 94:20200699. doi: 10.1259/bjr.20200699 33332981 PMC8011249

[42] Arevalo-Perez J Peck KK Young RJ Holodny AI Karimi S Lyo JK. Dynamic contrast-enhanced perfusion MRI and diffusion-weighted imaging in grading of gliomas. J Neuroimaging. (2015) 25:792–8. doi: 10.1111/jon.12239 PMC5525149 25867683

[43] Abe T Mizobuchi Y Nakajima K Otomi Y Irahara S Obama Y. Diagnosis of brain tumors using dynamic contrast-enhanced perfusion imaging with a short acquisition time. Springerplus. (2015) 4:88. doi: 10.1186/s40064-015-0861-6 25793147 PMC4359190

[44] Jung SC Yeom JA Kim J-H Ryoo I Kim SC Shin H. Glioma: Application of histogram analysis of pharmacokinetic parameters from T1-weighted dynamic contrast-enhanced MR imaging to tumor grading. AJNR Am J Neuroradiol. (2014) 35:1103–10. doi: 10.3174/ajnr.A3825 PMC7965150 24384119

[45] van Santwijk L Kouwenberg V Meijer F Smits M Henssen D. A systematic review and meta-analysis on the differentiation of glioma grade and mutational status by use of perfusion-based magnetic resonance imaging. Insights Imaging. (2022) 13:102. doi: 10.1186/s13244-022-01230-7 35670981 PMC9174367

[46] Okuchi S Rojas-Garcia A Ulyte A Lopez I Ušinskienė J Lewis M. Diagnostic accuracy of dynamic contrast-enhanced perfusion MRI in stratifying gliomas: A systematic review and meta-analysis. Cancer Med. (2019) 8:5564–73. doi: 10.1002/cam4.2369 PMC6745862 31389669

[47] Abrigo JM Fountain DM Provenzale JM Law EK Kwong JS Hart MG. Magnetic resonance perfusion for differentiating low-grade from high-grade gliomas at first presentation. Cochrane Database Syst Rev. (2018) 1:CD011551. doi: 10.1002/14651858.CD011551.pub2 29357120 PMC6491341

[48] Liang J Liu D Gao P Zhang D Chen H Shi C. Diagnostic values of DCE-MRI and DSC-MRI for differentiation between high-grade and low-grade gliomas: A comprehensive meta-analysis. Acad Radiol. (2018) 25:338–48. doi: 10.1016/j.acra.2017.10.001 29223713

[49] Zhang J Liu H Tong H Wang S Yang Y Liu G. Clinical applications of contrast-enhanced perfusion MRI techniques in gliomas: recent advances and current challenges. Contrast Media Mol Imaging. (2017) 2017:7064120. doi: 10.1155/2017/7064120 29097933 PMC5612612

[50] Santarosa C Castellano A Conte GM Cadioli M Iadanza A Terreni MR. Dynamic contrast-enhanced and dynamic susceptibility contrast perfusion MR imaging for glioma grading: Preliminary comparison of vessel compartment and permeability parameters using hotspot and histogram analysis. Eur J Radiol. (2016) 85:1147–56. doi: 10.1016/j.ejrad.2016.03.020 27161065

[51] Zhang N Zhang L Qiu B Meng L Wang X Hou BL. Correlation of volume transfer coefficient Ktrans with histopathologic grades of gliomas. J Magn Reson Imaging. (2012) 36:355–63. doi: 10.1002/jmri.23675 PMC3399966 22581762

[52] Awasthi R Rathore RKS Soni P Sahoo P Awasthi A Husain N. Discriminant analysis to classify glioma grading using dynamic contrast-enhanced MRI and immunohistochemical markers. Neuroradiology. (2012) 54:205–13. doi: 10.1007/s00234-011-0874-y 21541688

[53] Zhao M Guo L-L Huang N Wu Q Zhou L Zhao H. Quantitative analysis of permeability for glioma grading using dynamic contrast-enhanced magnetic resonance imaging. Oncol Lett. (2017) 14:5418–26. doi: 10.3892/ol.2017.6895 PMC5656018 29113174

[54] Sun S Qian H Li F Li Z Wu X. [Diagnostic value of combining permeability with T1 perfusion parameters in quantitative dynamic contrast-enhanced magnetic resonance imaging for glioma grading]. Zhongguo Yi Xue Ke Xue Yuan Xue Bao. (2015) 37:674–80. doi: 10.3881/j.issn.1000-503X.2015.06.007 26725390

[55] Louis DN Perry A Wesseling P Brat DJ Cree IA Figarella-Branger D. The 2021 WHO classification of tumors of the central nervous system: a summary. Neuro Oncol. (2021) 23:1231–51. doi: 10.1093/neuonc/noab106 PMC8328013 34185076

[56] Hu Y Chen Y Wang J Kang JJ Shen DD Jia ZZ. Non-invasive estimation of glioma IDH1 mutation and VEGF expression by histogram analysis of dynamic contrast-enhanced MRI. Front Oncol. (2020) 10:593102. doi: 10.3389/fonc.2020.593102 33425744 PMC7793903

[57] Keil VC Gielen GH Pintea B Baumgarten P Datsi A Hittatiya K. DCE-MRI in glioma, infiltration zone and healthy brain to assess angiogenesis: A biopsy study. Clin Neuroradiol. (2021) 31:1049–58. doi: 10.1007/s00062-021-01015-3 PMC8648693 33900414

[58] Ozturk K Soylu E Tolunay S Narter S Hakyemez B. Dynamic contrast-enhanced T1-weighted perfusion magnetic resonance imaging identifies glioblastoma immunohistochemical biomarkers via tumoral and peritumoral approach: A pilot study. World Neurosurg. (2019) 128:e195–208. doi: 10.1016/j.wneu.2019.04.089 31003026

[59] Li S-H Shen N-X Wu D Zhang J Zhang J-X Jiang J-J. A comparative study between tumor blood vessels and dynamic contrast-enhanced MRI for identifying isocitrate dehydrogenase gene 1 (IDH1) mutation status in glioma. Curr Med Sci. (2022) 42:650–7. doi: 10.1007/s11596-022-2563-y 35606665

[60] Park YW Ahn SS Park CJ Han K Kim EH Kang S-G. Diffusion and perfusion MRI may predict EGFR amplification and the TERT promoter mutation status of IDH-wildtype lower-grade gliomas. Eur Radiol. (2020) 30:6475–84. doi: 10.1007/s00330-020-07090-3 32785770

[61] Hu Y Zhang N Yu MH Zhou XJ Ge M Shen DD. Volume-based histogram analysis of dynamic contrast-enhanced MRI for estimation of gliomas IDH1 mutation status. Eur J Radiol. (2020) 131:109247. doi: 10.1016/j.ejrad.2020.109247 32891974

[62] Di N Cheng W Jiang X Liu X Zhou J Xie Q. Can dynamic contrast-enhanced MRI evaluate VEGF expression in brain glioma? An MRI-guided stereotactic biopsy study. J Neuroradiol. (2019) 46:186–92. doi: 10.1016/j.neurad.2018.04.008 29752976

[63] Di N Yao C Cheng W Ren Y Qu J Wang B. Correlation of dynamic contrast-enhanced MRI derived volume transfer constant with histological angiogenic markers in high-grade gliomas. J Med Imaging Radiat Oncol. (2018). doi: 10.1111/1754-9485.12701 29330968

[64] Beiko J Suki D Hess KR Fox BD Cheung V Cabral M. IDH1 mutant Malignant astrocytomas are more amenable to surgical resection and have a survival benefit associated with maximal surgical resection. Neuro Oncol. (2014) 16:81–91. doi: 10.1093/neuonc/not159 24305719 PMC3870823

[65] Wang X Cao M Chen H Ge J Suo S Zhou Y. Simplified perfusion fraction from diffusion-weighted imaging in preoperative prediction of IDH1 mutation in WHO grade II-III gliomas: comparison with dynamic contrast-enhanced and intravoxel incoherent motion MRI. Radiol Oncol. (2020) 54:301–10. doi: 10.2478/raon-2020-0037 PMC7409598 32559177

[66] Brendle C Hempel J-M Schittenhelm J Skardelly M Tabatabai G Bender B. Glioma grading and determination of IDH mutation status and ATRX loss by DCE and ASL perfusion. Clin Neuroradiol. (2018) 28:421–8. doi: 10.1007/s00062-017-0590-z 28488024

[67] Ahn SH Ahn SS Park YW Park CJ Lee S-K. Association of dynamic susceptibility contrast- and dynamic contrast-enhanced magnetic resonance imaging parameters with molecular marker status in lower-grade gliomas: A retrospective study. Neuroradiol J. (2023) 36:49–58. doi: 10.1177/19714009221098369 35532193 PMC9893160

[68] Chamberlain MC. Prognostic or predictive value of MGMT promoter methylation in gliomas depends on IDH1 mutation. Neurology. (2014) 82:2147–8. doi: 10.1212/01.wnl.0000451452.30826.6b 24912510

[69] Hilario A Hernandez-Lain A Sepulveda JM Lagares A Perez-Nuñez A Ramos A. Perfusion MRI grading diffuse gliomas: Impact of permeability parameters on molecular biomarkers and survival. Neurocirugia (Astur : Engl Ed). (2019) 30:11–8. doi: 10.1016/j.neucir.2018.06.004 30143443

[70] Ahn SS Shin N-Y Chang JH Kim SH Kim EH Kim DW. Prediction of methylguanine methyltransferase promoter methylation in glioblastoma using dynamic contrast-enhanced magnetic resonance and diffusion tensor imaging. J Neurosurg. (2014) 121:367–73. doi: 10.3171/2014.5.JNS132279 24949678

[71] Zhang H-W Lyu G-W He W-J Lei Y Lin F Wang M-Z. DSC and DCE histogram analyses of glioma biomarkers, including IDH, MGMT, and TERT, on differentiation and survival. Acad Radiol. (2020) 27:e263–71. doi: 10.1016/j.acra.2019.12.010 31983532

[72] Suh CH Kim HS Jung SC Park JE Choi CG Kim SJ. MRI as a diagnostic biomarker for differentiating primary central nervous system lymphoma from glioblastoma: A systematic review and meta-analysis. J Magn Reson Imaging. (2019) 50:560–72. doi: 10.1002/jmri.26602 30637843

[73] Chen X Xie T Fang J Xue W Tong H Kang H. Quantitative *in vivo* imaging of tissue factor expression in glioma using dynamic contrast-enhanced MRI derived parameters. Eur J Radiol. (2017) 93:236–42. doi: 10.1016/j.ejrad.2017.06.006 28668420

[74] Jung BC Arevalo-Perez J Lyo JK Holodny AI Karimi S Young RJ. Comparison of glioblastomas and brain metastases using dynamic contrast-enhanced perfusion MRI. J Neuroimaging. (2016) 26:240–6. doi: 10.1111/jon.12281 PMC4753138 26235208

[75] Zhang H-W Lyu G-W He W-J Lei Y Lin F Feng Y-N. Differential diagnosis of central lymphoma and high-grade glioma: dynamic contrast-enhanced histogram. Acta Radiol. (2020) 61:1221–7. doi: 10.1177/0284185119896519 31902220

[76] Parvaze PS Bhattacharjee R Verma YK Singh RK Yadav V Singh A. Quantification of Radiomics features of Peritumoral Vasogenic Edema extracted from fluid-attenuated inversion recovery images in glioblastoma and isolated brain metastasis, using T1-dynamic contrast-enhanced Perfusion analysis. NMR BioMed. (2023) 36:e4884. doi: 10.1002/nbm.4884 36453877

[77] Wang B Wang Z Jia Y Zhao P Han G Meng C. Water exchange detected by shutter speed dynamic contrast enhanced-MRI help distinguish solitary brain metastasis from glioblastoma. Eur J Radiol. (2022) 156:110526. doi: 10.1016/j.ejrad.2022.110526 36219917

[78] Lu S Wang S Gao Q Zhou M Li Y Cao P. Quantitative evaluation of diffusion and dynamic contrast-enhanced magnetic resonance imaging for differentiation between primary central nervous system lymphoma and glioblastoma. J Comput Assist Tomogr. (2017) 41:898–903. doi: 10.1097/RCT.0000000000000622 28806317

[79] Choi YS Lee H-J Ahn SS Chang JH Kang S-G Kim EH. Primary central nervous system lymphoma and atypical glioblastoma: differentiation using the initial area under the curve derived from dynamic contrast-enhanced MR and the apparent diffusion coefficient. Eur Radiol. (2017) 27:1344–51. doi: 10.1007/s00330-016-4484-2 27436023

[80] Xi Y-B Kang X-W Wang N Liu T-T Zhu Y-Q Cheng G. Differentiation of primary central nervous system lymphoma from high-grade glioma and brain metastasis using arterial spin labeling and dynamic contrast-enhanced magnetic resonance imaging. Eur J Radiol. (2019) 112:59–64. doi: 10.1016/j.ejrad.2019.01.008 30777220

[81] Kickingereder P Sahm F Wiestler B Roethke M Heiland S Schlemmer H-P. Evaluation of microvascular permeability with dynamic contrast-enhanced MRI for the differentiation of primary CNS lymphoma and glioblastoma: radiologic-pathologic correlation. AJNR Am J Neuroradiol. (2014) 35:1503–8. doi: 10.3174/ajnr.A3915 PMC7964431 24722313

[82] Lin X Lee M Buck O Woo KM Zhang Z Hatzoglou V. Diagnostic accuracy of T1-weighted dynamic contrast-enhanced-MRI and DWI-ADC for differentiation of glioblastoma and primary CNS lymphoma. AJNR Am J Neuroradiol. (2017) 38:485–91. doi: 10.3174/ajnr.A5023 PMC5352508 27932505

[83] Jin Y Peng H Peng J. Brain glioma localization diagnosis based on magnetic resonance imaging. World Neurosurg. (2021) 149:325–32. doi: 10.1016/j.wneu.2020.09.113 32992057

[84] Kang KM Choi SH Chul-Kee P Kim TM Park S-H Lee JH. Differentiation between glioblastoma and primary CNS lymphoma: application of DCE-MRI parameters based on arterial input function obtained from DSC-MRI. Eur Radiol. (2021) 31:9098–109. doi: 10.1007/s00330-021-08044-z 34003350

[85] Qiu J Tao Z-C Deng K-X Wang P Chen C-Y Xiao F. Diagnostic accuracy of dynamic contrast-enhanced magnetic resonance imaging for distinguishing pseudoprogression from glioma recurrence: a meta-analysis. Chin Med J(Engl). (2021) 134:2535–43. doi: 10.1097/CM9.0000000000001445 PMC8577681 34748524

[86] Khan MN Sharma AM Pitz M Loewen SK Quon H Poulin A. High-grade glioma management and response assessment-recent advances and current challenges. Curr Oncol. (2016) 23:e383–391. doi: 10.3747/co.23.3082 PMC4974045 27536188

[87] Artzi M Liberman G Nadav G Blumenthal DT Bokstein F Aizenstein O. Differentiation between treatment-related changes and progressive disease in patients with high grade brain tumors using support vector machine classification based on DCE MRI. J Neurooncol. (2016) 127:515–24. doi: 10.1007/s11060-016-2055-7 26754857

[88] Bisdas S Naegele T Ritz R Dimostheni A Pfannenberg C Reimold M. Distinguishing recurrent high-grade gliomas from radiation injury: a pilot study using dynamic contrast-enhanced MR imaging. Acad Radiol. (2011) 18:575–83. doi: 10.1016/j.acra.2011.01.018 21419671

[89] Zakhari N Taccone MS Torres CH Chakraborty S Sinclair J Woulfe J. Prospective comparative diagnostic accuracy evaluation of dynamic contrast-enhanced (DCE) vs. dynamic susceptibility contrast (DSC) MR perfusion in differentiating tumor recurrence from radiation necrosis in treated high-grade gliomas. J Magn Reson Imaging. (2019) 50:573–82. doi: 10.1002/jmri.26621 30614146

[90] Fahlström M Blomquist E Nyholm T Larsson E-M. Perfusion Magnetic Resonance Imaging Changes in Normal Appearing Brain Tissue after Radiotherapy in Glioblastoma Patients may Confound Longitudinal Evaluation of Treatment Response. Radiol Oncol. (2018) 52:143–51. doi: 10.2478/raon-2018-0022 PMC6043875 30018517

[91] Wang C Sun W Kirkpatrick J Chang Z Yin F-F. Assessment of concurrent stereotactic radiosurgery and bevacizumab treatment of recurrent Malignant gliomas using multi-modality MRI imaging and radiomics analysis. J Radiosurg SBRT. (2018) 5:171–81. PMC6018043 29988289

[92] Bressler I Ben Bashat D Buchsweiler Y Aizenstein O Limon D Bokestein F. Model-free dynamic contrast-enhanced MRI analysis: differentiation between active tumor and necrotic tissue in patients with glioblastoma. MAGMA. (2023) 36:33–42. doi: 10.1007/s10334-022-01045-z 36287282

[93] Thomas AA Arevalo-Perez J Kaley T Lyo J Peck KK Shi W. Dynamic contrast enhanced T1 MRI perfusion differentiates pseudoprogression from recurrent glioblastoma. J Neurooncol. (2015) 125:183–90. doi: 10.1007/s11060-015-1893-z PMC4726629 26275367

[94] Yun TJ Park C-K Kim TM Lee S-H Kim J-H Sohn C-H. Glioblastoma treated with concurrent radiation therapy and temozolomide chemotherapy: differentiation of true progression from pseudoprogression with quantitative dynamic contrast-enhanced MR imaging. Radiology. (2015) 274:830–40. doi: 10.1148/radiol.14132632 25333475

[95] Jing H Yan X Li J Qin D Zhang N Zhang H. The value of dynamic contrast-enhanced magnetic resonance imaging (DCE-MRI) in the differentiation of pseudoprogression and recurrence of intracranial gliomas. Contrast Media Mol Imaging. (2022) 2022:5680522. doi: 10.1155/2022/5680522 35935318 PMC9337951

[96] Jain R Ellika SK Scarpace L Schultz LR Rock JP Gutierrez J. Quantitative estimation of permeability surface-area product in astroglial brain tumors using perfusion CT and correlation with histopathologic grade. AJNR Am J Neuroradiol. (2008) 29:694–700. doi: 10.3174/ajnr.A0899 18202239 PMC7978188

[97] Jain R Griffith B Alotaibi F Zagzag D Fine H Golfinos J. Glioma angiogenesis and perfusion imaging: understanding the relationship between tumor blood volume and leakiness with increasing glioma grade. AJNR Am J Neuroradiol. (2015) 36:2030–5. doi: 10.3174/ajnr.A4405 PMC7964882 26206809

[98] Jensen RL Mumert ML Gillespie DL Kinney AY Schabel MC Salzman KL. Preoperative dynamic contrast-enhanced MRI correlates with molecular markers of hypoxia and vascularity in specific areas of intratumoral microenvironment and is predictive of patient outcome. Neuro Oncol. (2014) 16:280–91. doi: 10.1093/neuonc/not148 PMC3895375 24305704

[99] Koh TS Zeman V Darko J Lee TY Milosevic MF Haider M. The inclusion of capillary distribution in the adiabatic tissue homogeneity model of blood flow. Phys Med Biol. (2001) 46:1519–38. doi: 10.1088/0031-9155/46/5/313 11384068

[100] Schabel MC. A unified impulse response model for DCE-MRI. Magn Reson Med. (2012) 68:1632–46. doi: 10.1002/mrm.24162 22294448

[101] Lundemann M Munck Af Rosenschöld P Muhic A Larsen VA Poulsen HS Engelholm S-A. Feasibility of multi-parametric PET and MRI for prediction of tumour recurrence in patients with glioblastoma. Eur J Nucl Med Mol Imaging. (2019) 46:603–13. doi: 10.1007/s00259-018-4180-3 30276440

[102] Henriksen OM Hansen AE Muhic A Marner L Madsen K Møller S. Diagnostic yield of simultaneous dynamic contrast-enhanced magnetic resonance perfusion measurements and [18F]FET PET in patients with suspected recurrent anaplastic astrocytoma and glioblastoma. Eur J Nucl Med Mol Imaging. (2022) 49:4677–91. doi: 10.1007/s00259-022-05917-3 PMC9605929 35907033

[103] Larsen VA Simonsen HJ Law I Larsson HBW Hansen AE. Evaluation of dynamic contrast-enhanced T1-weighted perfusion MRI in the differentiation of tumor recurrence from radiation necrosis. Neuroradiology. (2013) 55:361–9. doi: 10.1007/s00234-012-1127-4 23262559

[104] Yeung TPC Kurdi M Wang Y Al-Khazraji B Morrison L Hoffman L. CT perfusion imaging as an early biomarker of differential response to stereotactic radiosurgery in C6 rat gliomas. PloS One. (2014) 9:e109781. doi: 10.1371/journal.pone.0109781 25329655 PMC4201465

[105] Li Z Zhao W He B Koh TS Li Y Zeng Y. Application of distributed parameter model to assessment of glioma IDH mutation status by dynamic contrast-enhanced magnetic resonance imaging. Contrast Media Mol Imaging. (2020) 2020:8843084. doi: 10.1155/2020/8843084 33299387 PMC7704178

[106] van den Bent MJ Gao Y Kerkhof M Kros JM Gorlia T van Zwieten K. Changes in the EGFR amplification and EGFRvIII expression between paired primary and recurrent glioblastomas. Neuro Oncol. (2015) 17:935–41. doi: 10.1093/neuonc/nov013 PMC5762005 25691693

[107] Nguyen TB Cron GO Mercier JF Foottit C Torres CH Chakraborty S. Diagnostic accuracy of dynamic contrast-enhanced MR imaging using a phase-derived vascular input function in the preoperative grading of gliomas. AJNR Am J Neuroradiol. (2012) 33:1539–45. doi: 10.3174/ajnr.A3012 PMC7966532 22442046

[108] Choi HS Kim AH Ahn SS Shin N Kim J Lee S-K. Glioma grading capability: comparisons among parameters from dynamic contrast-enhanced MRI and ADC value on DWI. Korean J Radiol. (2013) 14:487–92. doi: 10.3348/kjr.2013.14.3.487 PMC3655305 23690718

[109] Shukla-Dave A Obuchowski NA Chenevert TL Jambawalikar S Schwartz LH Malyarenko D. Quantitative imaging biomarkers alliance (QIBA) recommendations for improved precision of DWI and DCE-MRI derived biomarkers in multicenter oncology trials. J Magn Reson Imaging. (2019) 49:e101–21. doi: 10.1002/jmri.26518 PMC6526078 30451345

[110] Zagzag D Zhong H Scalzitti JM Laughner E Simons JW Semenza GL. Expression of hypoxia-inducible factor 1alpha in brain tumors: association with angiogenesis, invasion, and progression. Cancer. (2000) 88:2606–18. doi: 10.1002/(ISSN)1097-0142 10861440

[111] Xie Q Wu J Du Z Di N Yan R Pang H. DCE-MRI in human gliomas: A surrogate for assessment of invasive hypoxia marker HIF-1α Based on MRI-neuronavigation stereotactic biopsies. Acad Radiol. (2019) 26:179–87. doi: 10.1016/j.acra.2018.04.015 29754996

[112] Sourbron SP Buckley DL. On the scope and interpretation of the Tofts models for DCE-MRI. Magn Reson Med. (2011) 66:735–45. doi: 10.1002/mrm.22861 21384424

[113] Koh TS Hennedige TP Thng CH Hartono S Ng QS. Understanding K trans: a simulation study based on a multiple-pathway model. Phys Med Biol. (2017) 62:N297–319. doi: 10.1088/1361-6560/aa70c9 28467315

[114] Jacquez JA. Compartmental Analysis in Biology and Medicine. 2nd ed. Ann Arbor, MI: University of Michigan Press (1985).

[115] Anon. QIBA MR Biomarker Committee. MR DCE Quantification, Quantitative Imaging Biomarkers Alliance. Profile Stage: Public Comment. 2020–10-12 . Available online at: http://qibawiki.rsna.org/index.php/Profiles.

[116] Goh V Shastry M Engledow A Reston J Wellsted DM Peck J. Commercial software upgrades may significantly alter Perfusion CT parameter values in colorectal cancer. Eur Radiol. (2011) 21:744–9. doi: 10.1007/s00330-010-1967-4 20922392

[117] Andrews JL. Addressing overfitting and underfitting in Gaussian model-based clustering. Comput Stats Data Anal. (2018) 127:160–71. doi: 10.1016/j.csda.2018.05.015

